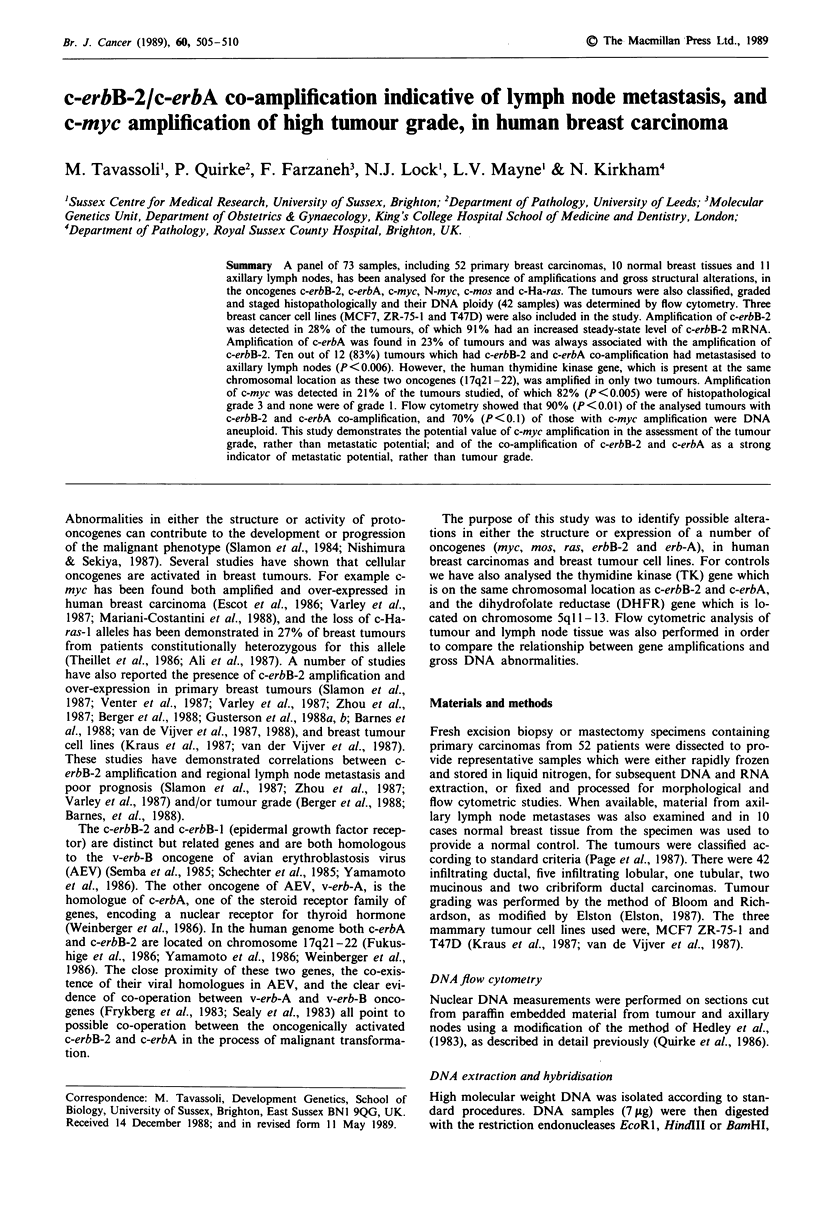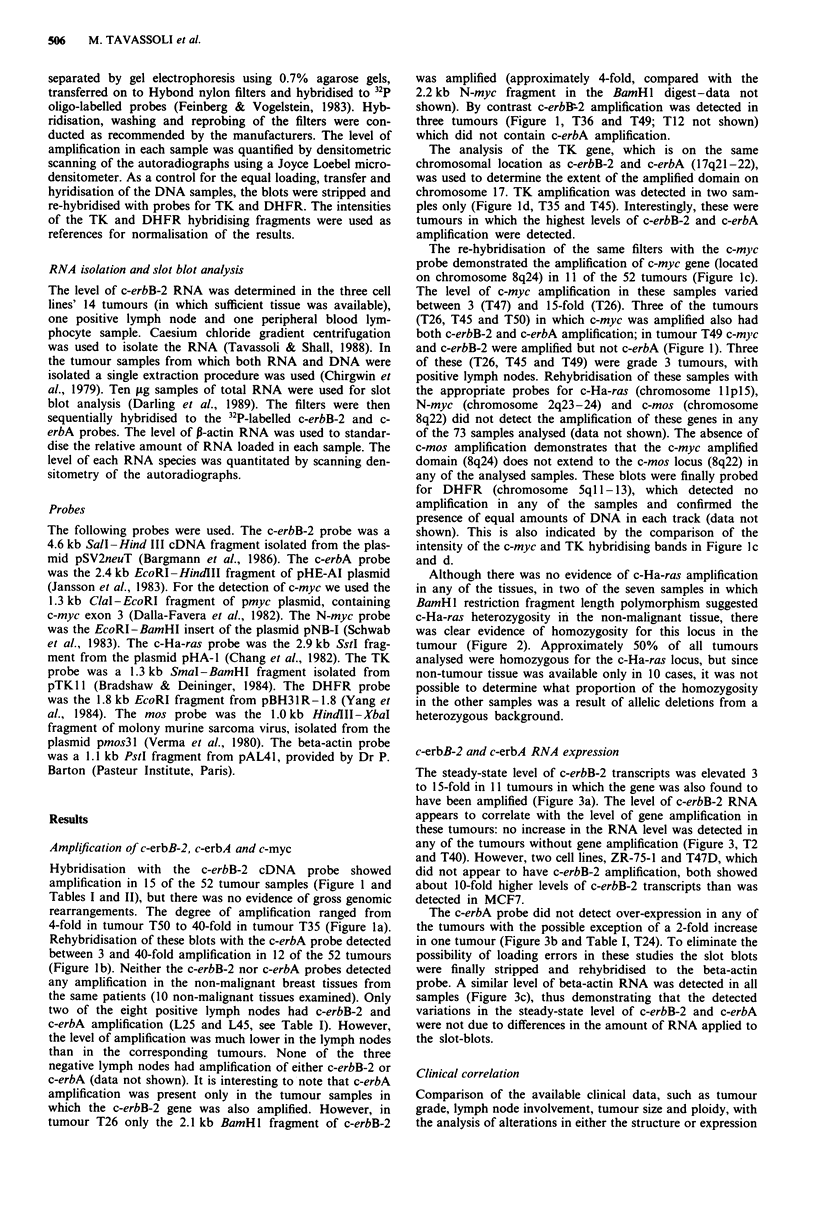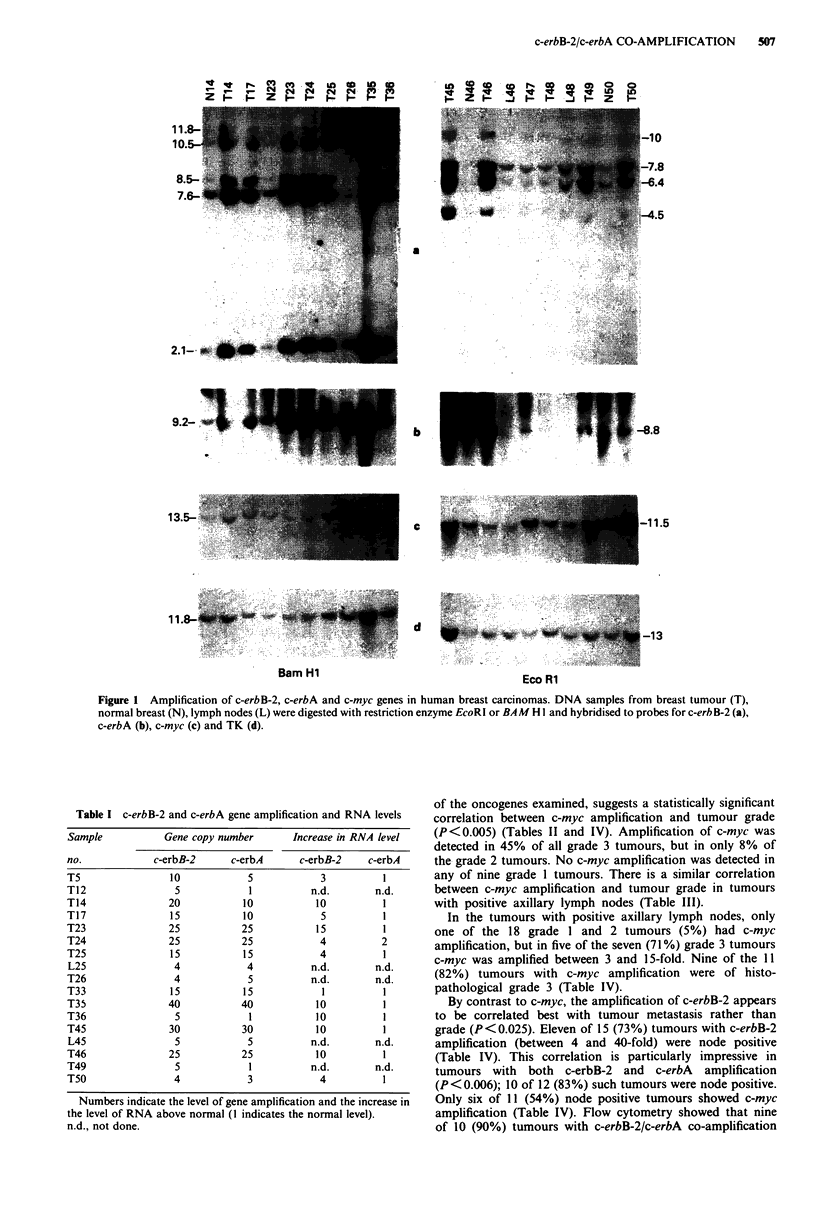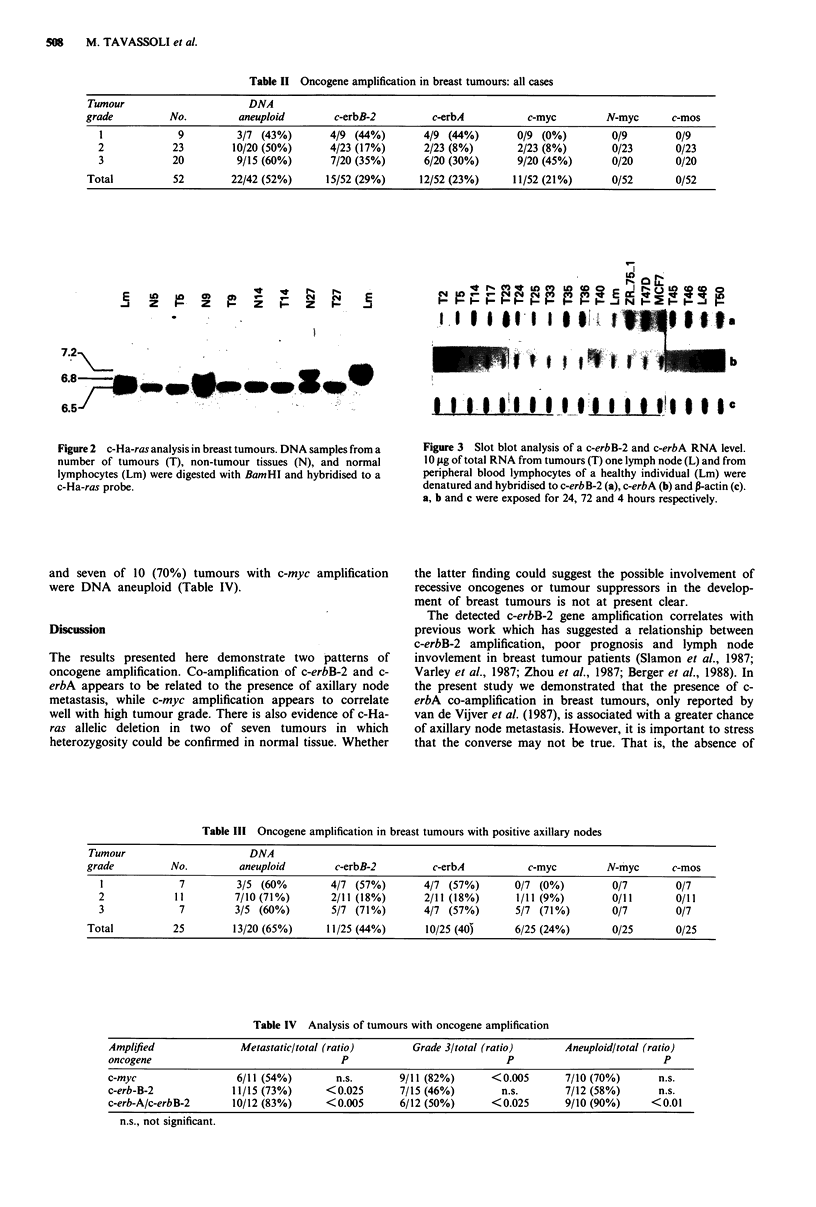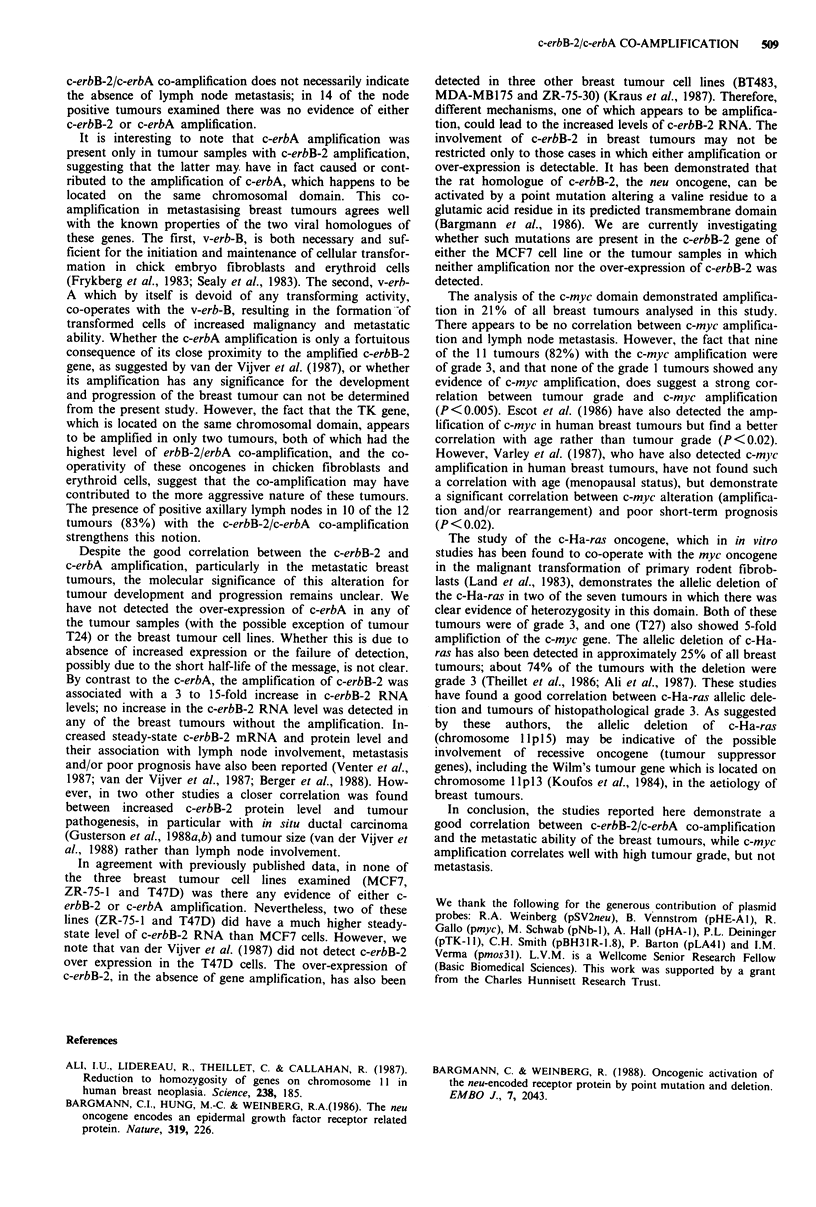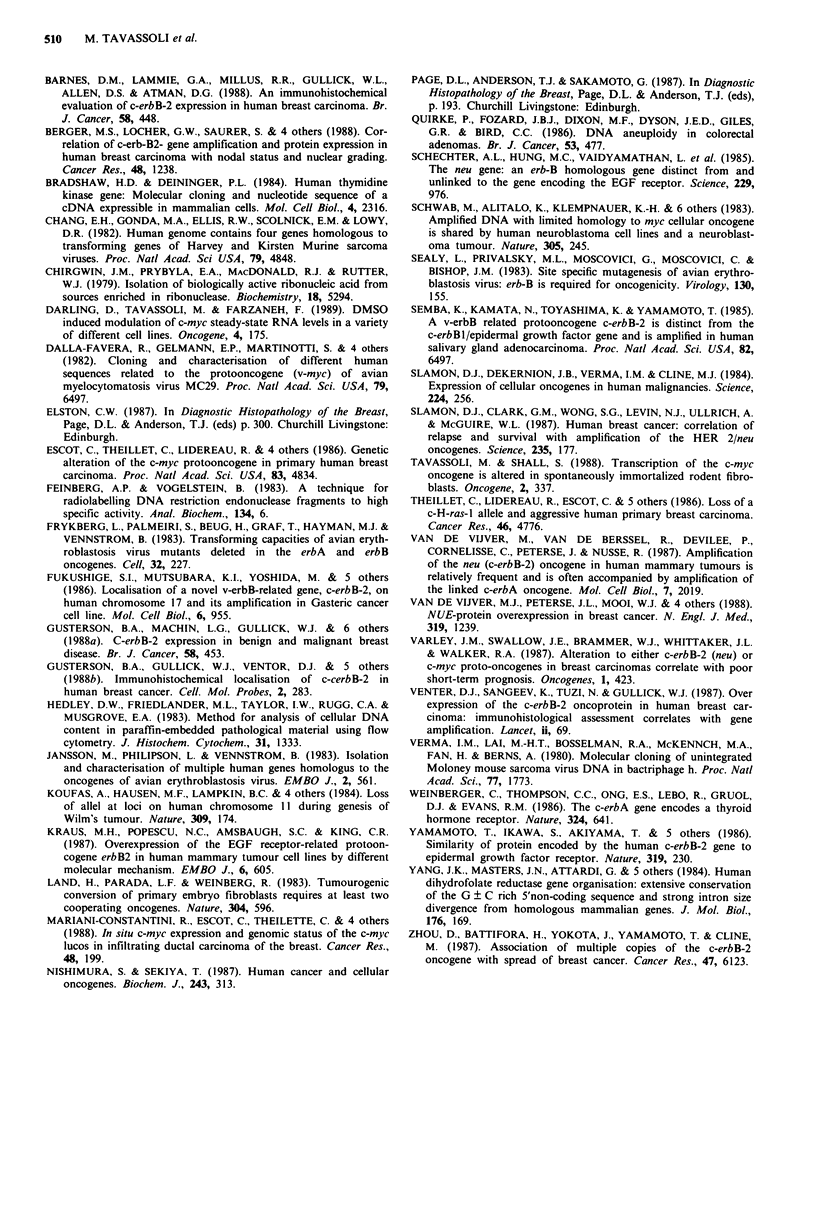# c-erbB-2/c-erbA co-amplification indicative of lymph node metastasis, and c-myc amplification of high tumour grade, in human breast carcinoma.

**DOI:** 10.1038/bjc.1989.303

**Published:** 1989-10

**Authors:** M. Tavassoli, P. Quirke, F. Farzaneh, N. J. Lock, L. V. Mayne, N. Kirkham

**Affiliations:** Sussex Centre for Medical Research, University of Sussex, Brighton, UK.

## Abstract

**Images:**


Br. J. Cancer (1989), 60, 505–510 © The Macmillan Press Ltd., 1989

### c-*erb*B-2/c-*erb*A co-amplification indicative of lymph node metastasis, and c-*myc* amplification of high tumour grade, in human breast carcinoma

M. Tavassoli[1], P. Quirke[2], F. Farzaneh[3], N.J. Lock[1], L.V. Mayne[1] & N. Kirkham[4]

[1]*Sussex Centre for Medical Research, University of Sussex, Brighton;* [2]*Department of Pathology, University of Leeds;* [3]*Molecular Genetics Unit, Department of Obstetrics & Gynaecology, King's College Hospital School of Medicine and Dentistry, London;* [4]*Department of Pathology, Royal Sussex County Hospital, Brighton, UK.*

**Summary** A panel of 73 samples, including 52 primary breast carcinomas, 10 normal breast tissues and 11 axillary lymph nodes, has been analysed for the presence of amplifications and gross structural alterations, in the oncogenes c-*erb*B-2, c-*erb*A, c-*myc*, N-*myc*, c-*mos* and c-Ha-*ras*. The tumours were also classified, graded and staged histopathologically and their DNA ploidy (42 samples) was determined by flow cytometry. Three breast cancer cell lines (MCF7, ZR-75-1 and T47D) were also included in the study. Amplification of c-*erb*B-2 was detected in 28% of the tumours, of which 91% had an increased steady-state level of c-*erb*B-2 mRNA. Amplification of c-*erb*A was found in 23% of tumours and was always associated with the amplification of c-*erb*B-2. Ten out of 12 (83%) tumours which had c-*erb*B-2 and c-*erb*A co-amplification had metastasised to axillary lymph nodes ($P<0.006$). However, the human thymidine kinase gene, which is present at the same chromosomal location as these two oncogenes (17q21–22), was amplified in only two tumours. Amplification of c-*myc* was detected in 21% of the tumours studied, of which 82% ($P<0.005$) were of histopathological grade 3 and none were of grade 1. Flow cytometry showed that 90% ($P<0.01$) of the analysed tumours with c-*erb*B-2 and c-*erb*A co-amplification, and 70% ($P<0.1$) of those with c-*myc* amplification were DNA aneuploid. This study demonstrates the potential value of c-*myc* amplification in the assessment of the tumour grade, rather than metastatic potential; and of the co-amplification of c-*erb*B-2 and c-*erb*A as a strong indicator of metastatic potential, rather than tumour grade.

Abnormalities in either the structure or activity of proto-oncogenes can contribute to the development or progression of the malignant phenotype (Slamon *et al.*, 1984; Nishimura & Sekiya, 1987). Several studies have shown that cellular oncogenes are activated in breast tumours. For example c-*myc* has been found both amplified and over-expressed in human breast carcinoma (Escot *et al.*, 1986; Varley *et al.*, 1987; Mariani-Costantini *et al.*, 1988), and the loss of c-Ha-*ras*-1 alleles has been demonstrated in 27% of breast tumours from patients constitutionally heterozygous for this allele (Theillet *et al.*, 1986; Ali *et al.*, 1987). A number of studies have also reported the presence of c-*erb*B-2 amplification and over-expression in primary breast tumours (Slamon *et al.*, 1987; Venter *et al.*, 1987; Varley *et al.*, 1987; Zhou *et al.*, 1987; Berger *et al.*, 1988; Gusterson *et al.*, 1988*a*, *b*; Barnes *et al.*, 1988; van de Vijver *et al.*, 1987, 1988), and breast tumour cell lines (Kraus *et al.*, 1987; van der Vijver *et al.*, 1987). These studies have demonstrated correlations between c-*erb*B-2 amplification and regional lymph node metastasis and poor prognosis (Slamon *et al.*, 1987; Zhou *et al.*, 1987; Varley *et al.*, 1987) and/or tumour grade (Berger *et al.*, 1988; Barnes, *et al.*, 1988).

The c-*erb*B-2 and c-*erb*B-1 (epidermal growth factor receptor) are distinct but related genes and are both homologous to the v-*erb*-B oncogene of avian erythroblastosis virus (AEV) (Semba *et al.*, 1985; Schechter *et al.*, 1985; Yamamoto *et al.*, 1986). The other oncogene of AEV, v-*erb*-A, is the homologue of c-*erb*A, one of the steroid receptor family of genes, encoding a nuclear receptor for thyroid hormone (Weinberger *et al.*, 1986). In the human genome both c-*erb*A and c-*erb*B-2 are located on chromosome 17q21–22 (Fukushige *et al.*, 1986; Yamamoto *et al.*, 1986; Weinberger *et al.*, 1986). The close proximity of these two genes, the co-existence of their viral homologues in AEV, and the clear evidence of co-operation between v-*erb*-A and v-*erb*-B oncogenes (Frykberg *et al.*, 1983; Sealy *et al.*, 1983) all point to possible co-operation between the oncogenically activated c-*erb*B-2 and c-*erb*A in the process of malignant transformation.

Correspondence: M. Tavassoli, Development Genetics, School of Biology, University of Sussex, Brighton, East Sussex BN1 9QG, UK.
Received 14 December 1988; and in revised form 11 May 1989.

The purpose of this study was to identify possible alterations in either the structure or expression of a number of oncogenes (*myc*, *mos*, *ras*, *erb*B-2 and *erb*-A), in human breast carcinomas and breast tumour cell lines. For controls we have also analysed the thymidine kinase (TK) gene which is on the same chromosomal location as c-*erb*B-2 and c-*erb*A, and the dihydrofolate reductase (DHFR) gene which is located on chromosome 5q11–13. Flow cytometric analysis of tumour and lymph node tissue was also performed in order to compare the relationship between gene amplifications and gross DNA abnormalities.

### Materials and methods

Fresh excision biopsy or mastectomy specimens containing primary carcinomas from 52 patients were dissected to provide representative samples which were either rapidly frozen and stored in liquid nitrogen, for subsequent DNA and RNA extraction, or fixed and processed for morphological and flow cytometric studies. When available, material from axillary lymph node metastases was also examined and in 10 cases normal breast tissue from the specimen was used to provide a normal control. The tumours were classified according to standard criteria (Page *et al.*, 1987). There were 42 infiltrating ductal, five infiltrating lobular, one tubular, two mucinous and two cribriform ductal carcinomas. Tumour grading was performed by the method of Bloom and Richardson, as modified by Elston (Elston, 1987). The three mammary tumour cell lines used were, MCF7 ZR-75-1 and T47D (Kraus *et al.*, 1987; van de Vijver *et al.*, 1987).

### *DNA flow cytometry*

Nuclear DNA measurements were performed on sections cut from paraffin embedded material from tumour and axillary nodes using a modification of the method of Hedley *et al.*, (1983), as described in detail previously (Quirke *et al.*, 1986).

### *DNA extraction and hybridisation*

High molecular weight DNA was isolated according to standard procedures. DNA samples (7 μg) were then digested with the restriction endonucleases *Eco*R1, *Hind*III or *Bam*HI, separated by gel electrophoresis using 0.7% agarose gels, transferred on to Hybond nylon filters and hybridised to $^{32}P$ oligo-labelled probes (Feinberg & Vogelstein, 1983). Hybridisation, washing and reprobing of the filters were conducted as recommended by the manufacturers. The level of amplification in each sample was quantified by densitometric scanning of the autoradiographs using a Joyce Loebel microdensitometer. As a control for the equal loading, transfer and hyridisation of the DNA samples, the blots were stripped and re-hybridised with probes for TK and DHFR. The intensities of the TK and DHFR hybridising fragments were used as references for normalisation of the results.

*RNA isolation and slot blot analysis*

The level of c-*erb*B-2 RNA was determined in the three cell lines' 14 tumours (in which sufficient tissue was available), one positive lymph node and one peripheral blood lymphocyte sample. Caesium chloride gradient centrifugation was used to isolate the RNA (Tavassoli & Shall, 1988). In the tumour samples from which both RNA and DNA were isolated a single extraction procedure was used (Chirgwin *et al.*, 1979). Ten μg samples of total RNA were used for slot blot analysis (Darling *et al.*, 1989). The filters were then sequentially hybridised to the $^{32}P$-labelled c-*erb*B-2 and c-*erb*A probes. The level of β-actin RNA was used to standardise the relative amount of RNA loaded in each sample. The level of each RNA species was quantitated by scanning densitometry of the autoradiographs.

*Probes*

The following probes were used. The c-*erb*B-2 probe was a 4.6 kb *Sal*I–*Hind* III cDNA fragment isolated from the plasmid pSV2*neu*T (Bargmann *et al.*, 1986). The c-*erb*A probe was the 2.4 kb *Eco*RI–*Hind*III fragment of pHE-AI plasmid (Jansson *et al.*, 1983). For the detection of c-*myc* we used the 1.3 kb *Cla*I–*Eco*RI fragment of p*myc* plasmid, containing c-*myc* exon 3 (Dalla-Favera *et al.*, 1982). The N-*myc* probe was the *Eco*RI–*Bam*HI insert of the plasmid pNB-I (Schwab *et al.*, 1983). The c-Ha-*ras* probe was the 2.9 kb *Sst*I fragment from the plasmid pHA-1 (Chang *et al.*, 1982). The TK probe was a 1.3 kb *Sma*I–*Bam*HI fragment isolated from pTK11 (Bradshaw & Deininger, 1984). The DHFR probe was the 1.8 kb *Eco*RI fragment from pBH31R–1.8 (Yang *et al.*, 1984). The *mos* probe was the 1.0 kb *Hind*III–*Xba*I fragment of molony murine sarcoma virus, isolated from the plasmid p*mos*31 (Verma *et al.*, 1980). The beta-actin probe was a 1.1 kb *Pst*I fragment from pAL41, provided by Dr P. Barton (Pasteur Institute, Paris).

### Results

*Amplification of* c-erbB-2, c-erbA *and* c-myc

Hybridisation with the c-*erb*B-2 cDNA probe showed amplification in 15 of the 52 tumour samples (Figure 1 and Tables I and II), but there was no evidence of gross genomic rearrangements. The degree of amplification ranged from 4-fold in tumour T50 to 40-fold in tumour T35 (Figure 1a). Rehybridisation of these blots with the c-*erb*A probe detected between 3 and 40-fold amplification in 12 of the 52 tumours (Figure 1b). Neither the c-*erb*B-2 nor c-*erb*A probes detected any amplification in the non-malignant breast tissues from the same patients (10 non-malignant tissues examined). Only two of the eight positive lymph nodes had c-*erb*B-2 and c-*erb*A amplification (L25 and L45, see Table I). However, the level of amplification was much lower in the lymph nodes than in the corresponding tumours. None of the three negative lymph nodes had amplification of either c-*erb*B-2 or c-*erb*A (data not shown). It is interesting to note that c-*erb*A amplification was present only in the tumour samples in which the c-*erb*B-2 gene was also amplified. However, in tumour T26 only the 2.1 kb *Bam*HI fragment of c-*erb*B-2 was amplified (approximately 4-fold, compared with the 2.2 kb N-*myc* fragment in the *Bam*HI digest–data not shown). By contrast c-*erb*B-2 amplification was detected in three tumours (Figure 1, T36 and T49; T12 not shown) which did not contain c-*erb*A amplification.

The analysis of the TK gene, which is on the same chromosomal location as c-*erb*B-2 and c-*erb*A (17q21–22), was used to determine the extent of the amplified domain on chromosome 17. TK amplification was detected in two samples only (Figure 1d, T35 and T45). Interestingly, these were tumours in which the highest levels of c-*erb*B-2 and c-*erb*A amplification were detected.

The re-hybridisation of the same filters with the c-*myc* probe demonstrated the amplification of c-*myc* gene (located on chromosome 8q24) in 11 of the 52 tumours (Figure 1c). The level of c-*myc* amplification in these samples varied between 3 (T47) and 15-fold (T26). Three of the tumours (T26, T45 and T50) in which c-*myc* was amplified also had both c-*erb*B-2 and c-*erb*A amplification; in tumour T49 c-*myc* and c-*erb*B-2 were amplified but not c-*erb*A (Figure 1). Three of these (T26, T45 and T49) were grade 3 tumours, with positive lymph nodes. Rehybridisation of these samples with the appropriate probes for c-Ha-*ras* (chromosome 11p15), N-*myc* (chromosome 2q23–24) and c-*mos* (chromosome 8q22) did not detect the amplification of these genes in any of the 73 samples analysed (data not shown). The absence of c-*mos* amplification demonstrates that the c-*myc* amplified domain (8q24) does not extend to the c-*mos* locus (8q22) in any of the analysed samples. These blots were finally probed for DHFR (chromosome 5q11–13), which detected no amplification in any of the samples and confirmed the presence of equal amounts of DNA in each track (data not shown). This is also indicated by the comparison of the intensity of the c-*myc* and TK hybridising bands in Figure 1c and d.

Although there was no evidence of c-Ha-*ras* amplification in any of the tissues, in two of the seven samples in which *Bam*HI restriction fragment length polymorphism suggested c-Ha-*ras* heterozygosity in the non-malignant tissue, there was clear evidence of homozygosity for this locus in the tumour (Figure 2). Approximately 50% of all tumours analysed were homozygous for the c-Ha-*ras* locus, but since non-tumour tissue was available only in 10 cases, it was not possible to determine what proportion of the homozygosity in the other samples was a result of allelic deletions from a heterozygous background.

c-erb*B-2 and* c-erb*A RNA expression*

The steady-state level of c-*erb*B-2 transcripts was elevated 3 to 15-fold in 11 tumours in which the gene was also found to have been amplified (Figure 3a). The level of c-*erb*B-2 RNA appears to correlate with the level of gene amplification in these tumours: no increase in the RNA level was detected in any of the tumours without gene amplification (Figure 3, T2 and T40). However, two cell lines, ZR-75-1 and T47D, which did not appear to have c-*erb*B-2 amplification, both showed about 10-fold higher levels of c-*erb*B-2 transcripts than was detected in MCF7.

The c-*erb*A probe did not detect over-expression in any of the tumours with the possible exception of a 2-fold increase in one tumour (Figure 3b and Table I, T24). To eliminate the possibility of loading errors in these studies the slot blots were finally stripped and rehybridised to the beta-actin probe. A similar level of beta-actin RNA was detected in all samples (Figure 3c), thus demonstrating that the detected variations in the steady-state level of c-*erb*B-2 and c-*erb*A were not due to differences in the amount of RNA applied to the slot-blots.

*Clinical correlation*

Comparison of the available clinical data, such as tumour grade, lymph node involvement, tumour size and ploidy, with the analysis of alterations in either the structure or expression

**Figure 1** Amplification of c-*erb*B-2, c-*erb*A and c-*myc* genes in human breast carcinomas. DNA samples from breast tumour (T), normal breast (N), lymph nodes (L) were digested with restriction enzyme *Eco*RI or *BAM* H1 and hybridised to probes for c-*erb*B-2 (**a**), c-*erb*A (**b**), c-*myc* (**c**) and TK (**d**).

**Table I** c-*erb*B-2 and c-*erb*A gene amplification and RNA levels

| *Sample* | *Gene copy number* | | *Increase in RNA level* | |
|---|---|---|---|---|
| *no.* | c-erb*B-2* | c-erb*A* | c-erb*B-2* | c-erb*A* |
| T5 | 10 | 5 | 3 | 1 |
| T12 | 5 | 1 | n.d. | n.d. |
| T14 | 20 | 10 | 10 | 1 |
| T17 | 15 | 10 | 5 | 1 |
| T23 | 25 | 25 | 15 | 1 |
| T24 | 25 | 25 | 4 | 2 |
| T25 | 15 | 15 | 4 | 1 |
| L25 | 4 | 4 | n.d. | n.d. |
| T26 | 4 | 5 | n.d. | n.d. |
| T33 | 15 | 15 | 1 | 1 |
| T35 | 40 | 40 | 10 | 1 |
| T36 | 5 | 1 | 10 | 1 |
| T45 | 30 | 30 | 10 | 1 |
| L45 | 5 | 5 | n.d. | n.d. |
| T46 | 25 | 25 | 10 | 1 |
| T49 | 5 | 1 | n.d. | n.d. |
| T50 | 4 | 3 | 4 | 1 |

Numbers indicate the level of gene amplification and the increase in the level of RNA above normal (1 indicates the normal level).
n.d., not done.

of the oncogenes examined, suggests a statistically significant correlation between c-*myc* amplification and tumour grade ($P < 0.005$) (Tables II and IV). Amplification of c-*myc* was detected in 45% of all grade 3 tumours, but in only 8% of the grade 2 tumours. No c-*myc* amplification was detected in any of nine grade 1 tumours. There is a similar correlation between c-*myc* amplification and tumour grade in tumours with positive axillary lymph nodes (Table III).

In the tumours with positive axillary lymph nodes, only one of the 18 grade 1 and 2 tumours (5%) had c-*myc* amplification, but in five of the seven (71%) grade 3 tumours c-*myc* was amplified between 3 and 15-fold. Nine of the 11 (82%) tumours with c-*myc* amplification were of histopathological grade 3 (Table IV).

By contrast to c-*myc*, the amplification of c-*erb*B-2 appears to be correlated best with tumour metastasis rather than grade ($P < 0.025$). Eleven of 15 (73%) tumours with c-*erb*B-2 amplification (between 4 and 40-fold) were node positive (Table IV). This correlation is particularly impressive in tumours with both c-erbB-2 and c-*erb*A amplification ($P < 0.006$); 10 of 12 (83%) such tumours were node positive. Only six of 11 (54%) node positive tumours showed c-*myc* amplification (Table IV). Flow cytometry showed that nine of 10 (90%) tumours with c-*erb*B-2/c-*erb*A co-amplification

**Table II** Oncogene amplification in breast tumours: all cases

| *Tumour grade* | *No.* | *DNA aneuploid* | *c*-erb*B-2* | *c*-erb*A* | *c*-myc | *N*-myc | *c*-mos |
|---|---|---|---|---|---|---|---|
| 1 | 9 | 3/7 (43%) | 4/9 (44%) | 4/9 (44%) | 0/9 (0%) | 0/9 | 0/9 |
| 2 | 23 | 10/20 (50%) | 4/23 (17%) | 2/23 (8%) | 2/23 (8%) | 0/23 | 0/23 |
| 3 | 20 | 9/15 (60%) | 7/20 (35%) | 6/20 (30%) | 9/20 (45%) | 0/20 | 0/20 |
| Total | 52 | 22/42 (52%) | 15/52 (29%) | 12/52 (23%) | 11/52 (21%) | 0/52 | 0/52 |

**Figure 2** c-Ha-*ras* analysis in breast tumours. DNA samples from a number of tumours (T), non-tumour tissues (N), and normal lymphocytes (Lm) were digested with *Bam*HI and hybridised to a c-Ha-*ras* probe.

**Figure 3** Slot blot analysis of a c-*erb*B-2 and c-*erb*A RNA level. 10 μg of total RNA from tumours (T) one lymph node (L) and from peripheral blood lymphocytes of a healthy individual (Lm) were denatured and hybridised to c-*erb*B-2 (**a**), c-*erb*A (**b**) and β-actin (**c**). **a**, **b** and **c** were exposed for 24, 72 and 4 hours respectively.

and seven of 10 (70%) tumours with c-*myc* amplification were DNA aneuploid (Table IV).

### Discussion

The results presented here demonstrate two patterns of oncogene amplification. Co-amplification of c-*erb*B-2 and c-*erb*A appears to be related to the presence of axillary node metastasis, while c-*myc* amplification appears to correlate well with high tumour grade. There is also evidence of c-Ha-*ras* allelic deletion in two of seven tumours in which heterozygosity could be confirmed in normal tissue. Whether the latter finding could suggest the possible involvement of recessive oncogenes or tumour suppressors in the development of breast tumours is not at present clear.

The detected c-*erb*B-2 gene amplification correlates with previous work which has suggested a relationship between c-*erb*B-2 amplification, poor prognosis and lymph node invovlement in breast tumour patients (Slamon *et al.*, 1987; Varley *et al.*, 1987; Zhou *et al.*, 1987; Berger *et al.*, 1988). In the present study we demonstrated that the presence of c-*erb*A co-amplification in breast tumours, only reported by van de Vijver *et al.* (1987), is associated with a greater chance of axillary node metastasis. However, it is important to stress that the converse may not be true. That is, the absence of

**Table III** Oncogene amplification in breast tumours with positive axillary nodes

| *Tumour grade* | *No.* | *DNA aneuploid* | *c*-erb*B-2* | *c*-erb*A* | *c*-myc | *N*-myc | *c*-mos |
|---|---|---|---|---|---|---|---|
| 1 | 7 | 3/5 (60% | 4/7 (57%) | 4/7 (57%) | 0/7 (0%) | 0/7 | 0/7 |
| 2 | 11 | 7/10 (71%) | 2/11 (18%) | 2/11 (18%) | 1/11 (9%) | 0/11 | 0/11 |
| 3 | 7 | 3/5 (60%) | 5/7 (71%) | 4/7 (57%) | 5/7 (71%) | 0/7 | 0/7 |
| Total | 25 | 13/20 (65%) | 11/25 (44%) | 10/25 (40) | 6/25 (24%) | 0/25 | 0/25 |

**Table IV** Analysis of tumours with oncogene amplification

| *Amplified oncogene* | *Metastatic/total (ratio)* | *P* | *Grade 3/total (ratio)* | *P* | *Aneuploid/total (ratio)* | *P* |
|---|---|---|---|---|---|---|
| c-*myc* | 6/11 (54%) | n.s. | 9/11 (82%) | <0.005 | 7/10 (70%) | n.s. |
| c-*erb*-B-2 | 11/15 (73%) | <0.025 | 7/15 (46%) | n.s. | 7/12 (58%) | n.s. |
| c-*erb*-A/c-*erb*B-2 | 10/12 (83%) | <0.005 | 6/12 (50%) | <0.025 | 9/10 (90%) | <0.01 |

n.s., not significant.

c-*erb*B-2/c-*erb*A co-amplification does not necessarily indicate the absence of lymph node metastasis; in 14 of the node positive tumours examined there was no evidence of either c-*erb*B-2 or c-*erb*A amplification.

It is interesting to note that c-*erb*A amplification was present only in tumour samples with c-*erb*B-2 amplification, suggesting that the latter may have in fact caused or contributed to the amplification of c-*erb*A, which happens to be located on the same chromosomal domain. This co-amplification in metastasising breast tumours agrees well with the known properties of the two viral homologues of these genes. The first, v-*erb*-B, is both necessary and sufficient for the initiation and maintenance of cellular transformation in chick embryo fibroblasts and erythroid cells (Frykberg *et al.*, 1983; Sealy *et al.*, 1983). The second, v-*erb*-A which by itself is devoid of any transforming activity, co-operates with the v-*erb*-B, resulting in the formation of transformed cells of increased malignancy and metastatic ability. Whether the c-*erb*A amplification is only a fortuitous consequence of its close proximity to the amplified c-*erb*B-2 gene, as suggested by van der Vijver *et al.* (1987), or whether its amplification has any significance for the development and progression of the breast tumour can not be determined from the present study. However, the fact that the TK gene, which is located on the same chromosomal domain, appears to be amplified in only two tumours, both of which had the highest level of *erb*B-2/*erb*A co-amplification, and the co-operativity of these oncogenes in chicken fibroblasts and erythroid cells, suggest that the co-amplification may have contributed to the more aggressive nature of these tumours. The presence of positive axillary lymph nodes in 10 of the 12 tumours (83%) with the c-*erb*B-2/c-*erb*A co-amplification strengthens this notion.

Despite the good correlation between the c-*erb*B-2 and c-*erb*A amplification, particularly in the metastatic breast tumours, the molecular significance of this alteration for tumour development and progression remains unclear. We have not detected the over-expression of c-*erb*A in any of the tumour samples (with the possible exception of tumour T24) or the breast tumour cell lines. Whether this is due to absence of increased expression or the failure of detection, possibly due to the short half-life of the message, is not clear. By contrast to the c-*erb*A, the amplification of c-*erb*B-2 was associated with a 3 to 15-fold increase in c-*erb*B-2 RNA levels; no increase in the c-*erb*B-2 RNA level was detected in any of the breast tumours without the amplification. Increased steady-state c-*erb*B-2 mRNA and protein level and their association with lymph node involvement, metastasis and/or poor prognosis have also been reported (Venter *et al.*, 1987; van der Vijver *et al.*, 1987; Berger *et al.*, 1988). However, in two other studies a closer correlation was found between increased c-*erb*B-2 protein level and tumour pathogenesis, in particular with *in situ* ductal carcinoma (Gusterson *et al.*, 1988*a,b*) and tumour size (van der Vijver *et al.*, 1988) rather than lymph node involvement.

In agreement with previously published data, in none of the three breast tumour cell lines examined (MCF7, ZR-75-1 and T47D) was there any evidence of either c-*erb*B-2 or c-*erb*A amplification. Nevertheless, two of these lines (ZR-75-1 and T47D) did have a much higher steady-state level of c-*erb*B-2 RNA than MCF7 cells. However, we note that van der Vijver *et al.* (1987) did not detect c-*erb*B-2 over expression in the T47D cells. The over-expression of c-*erb*B-2, in the absence of gene amplification, has also been detected in three other breast tumour cell lines (BT483, MDA-MB175 and ZR-75-30) (Kraus *et al.*, 1987). Therefore, different mechanisms, one of which appears to be amplification, could lead to the increased levels of c-*erb*B-2 RNA. The involvement of c-*erb*B-2 in breast tumours may not be restricted only to those cases in which either amplification or over-expression is detectable. It has been demonstrated that the rat homologue of c-*erb*B-2, the *neu* oncogene, can be activated by a point mutation altering a valine residue to a glutamic acid residue in its predicted transmembrane domain (Bargmann *et al.*, 1986). We are currently investigating whether such mutations are present in the c-*erb*B-2 gene of either the MCF7 cell line or the tumour samples in which neither amplification nor the over-expression of c-*erb*B-2 was detected.

The analysis of the c-*myc* domain demonstrated amplification in 21% of all breast tumours analysed in this study. There appears to be no correlation between c-*myc* amplification and lymph node metastasis. However, the fact that nine of the 11 tumours (82%) with the c-*myc* amplification were of grade 3, and that none of the grade 1 tumours showed any evidence of c-*myc* amplification, does suggest a strong correlation between tumour grade and c-*myc* amplification ($P<0.005$). Escot *et al.* (1986) have also detected the amplification of c-*myc* in human breast tumours but find a better correlation with age rather than tumour grade ($P<0.02$). However, Varley *et al.* (1987), who have also detected c-*myc* amplification in human breast tumours, have not found such a correlation with age (menopausal status), but demonstrate a significant correlation between c-*myc* alteration (amplification and/or rearrangement) and poor short-term prognosis ($P<0.02$).

The study of the c-Ha-*ras* oncogene, which in *in vitro* studies has been found to co-operate with the *myc* oncogene in the malignant transformation of primary rodent fibroblasts (Land *et al.*, 1983), demonstrates the allelic deletion of the c-Ha-*ras* in two of the seven tumours in which there was clear evidence of heterozygosity in this domain. Both of these tumours were of grade 3, and one (T27) also showed 5-fold amplifiction of the c-*myc* gene. The allelic deletion of c-Ha-*ras* has also been detected in approximately 25% of all breast tumours; about 74% of the tumours with the deletion were grade 3 (Theillet *et al.*, 1986; Ali *et al.*, 1987). These studies have found a good correlation between c-Ha-*ras* allelic deletion and tumours of histopathological grade 3. As suggested by these authors, the allelic deletion of c-Ha-*ras* (chromosome 11p15) may be indicative of the possible involvement of recessive oncogene (tumour suppressor genes), including the Wilm's tumour gene which is located on chromosome 11p13 (Koufos *et al.*, 1984), in the aetiology of breast tumours.

In conclusion, the studies reported here demonstrate a good correlation between c-*erb*B-2/c-*erb*A co-amplification and the metastatic ability of the breast tumours, while c-*myc* amplification correlates well with high tumour grade, but not metastasis.

We thank the following for the generous contribution of plasmid probes: R.A. Weinberg (pSV2*neu*), B. Vennstrom (pHE-A1), R. Gallo (p*myc*), M. Schwab (pNb-1), A. Hall (pHA-1), P.L. Deininger (pTK-11), C.H. Smith (pBH31R-1.8), P. Barton (pLA41) and I.M. Verma (p*mos*31). L.V.M. is a Wellcome Senior Research Fellow (Basic Biomedical Sciences). This work was supported by a grant from the Charles Hunnisett Research Trust.

### References

ALI, I.U., LIDEREAU, R., THEILLET, C. & CALLAHAN, R. (1987). Reduction to homozygosity of genes on chromosome 11 in human breast neoplasia. *Science*, **238**, 185.

BARGMANN, C.I., HUNG, M.-C. & WEINBERG, R.A.(1986). The *neu* oncogene encodes an epidermal growth factor receptor related protein. *Nature*, **319**, 226.

BARGMANN, C. & WEINBERG, R. (1988). Oncogenic activation of the *neu*-encoded receptor protein by point mutation and deletion. *EMBO J.*, **7**, 2043.

BARNES, D.M., LAMMIE, G.A., MILLUS, R.R., GULLICK, W.L., ALLEN, D.S. & ATMAN, D.G. (1988). An immunohistochemical evaluation of c-*erb*B-2 expression in human breast carcinoma. *Br. J. Cancer*, **58**, 448.

BERGER, M.S., LOCHER, G.W., SAURER, S. & 4 others (1988). Correlation of c-erb-B2- gene amplification and protein expression in human breast carcinoma with nodal status and nuclear grading. *Cancer Res.*, **48**, 1238.

BRADSHAW, H.D. & DEININGER, P.L. (1984). Human thymidine kinase gene: Molecular cloning and nucleotide sequence of a cDNA expressible in mammalian cells. *Mol. Cell Biol.*, **4**, 2316.

CHANG, E.H., GONDA, M.A., ELLIS, R.W., SCOLNICK, E.M. & LOWY, D.R. (1982). Human genome contains four genes homologous to transforming genes of Harvey and Kirsten Murine sarcoma viruses. *Proc. Natl Acad. Sci USA*, **79**, 4848.

CHIRGWIN, J.M., PRYBYLA, E.A., MACDONALD, R.J. & RUTTER, W.J. (1979). Isolation of biologically active ribonucleic acid from sources enriched in ribonuclease. *Biochemistry*, **18**, 5294.

DARLING, D., TAVASSOLI, M. & FARZANEH, F. (1989). DMSO induced modulation of c-*myc* steady-state RNA levels in a variety of different cell lines. *Oncogene*, **4**, 175.

DALLA-FAVERA, R., GELMANN, E.P., MARTINOTTI, S. & 4 others (1982). Cloning and characterisation of different human sequences related to the protooncogene (v-*myc*) of avian myelocytomatosis virus MC29. *Proc. Natl Acad. Sci. USA*, **79**, 6497.

ELSTON, C.W. (1987). In *Diagnostic Histopathology of the Breast*, Page, D.L. & Anderson, T.J. (eds) p. 300. Churchill Livingstone: Edinburgh.

ESCOT, C., THEILLET, C., LIDEREAU, R. & 4 others (1986). Genetic alteration of the c-*myc* protooncogene in primary human breast carcinoma. *Proc. Natl Acad. Sci. USA*, **83**, 4834.

FEINBERG, A.P. & VOGELSTEIN, B. (1983). A technique for radiolabelling DNA restriction endonuclease fragments to high specific activity. *Anal. Biochem.*, **134**, 6.

FRYKBERG, L., PALMEIRI, S., BEUG, H., GRAF, T., HAYMAN, M.J. & VENNSTROM, B. (1983). Transforming capacities of avian erythroblastosis virus mutants deleted in the *erb*A and *erb*B oncogenes. *Cell*, **32**, 227.

FUKUSHIGE, S.I., MUTSUBARA, K.I., YOSHIDA, M. & 5 others (1986). Localisation of a novel v-erbB-related gene, c-*erb*B-2, on human chromosome 17 and its amplification in Gasteric cancer cell line. *Mol. Cell Biol.*, **6**, 955.

GUSTERSON, B.A., MACHIN, L.G., GULLICK, W.J. & 6 others (1988*a*). C-*erb*B-2 expression in benign and malignant breast disease. *Br. J. Cancer*, **58**, 453.

GUSTERSON, B.A., GULLICK, W.J., VENTOR, D.J. & 5 others (1988*b*). Immunohistochemical localisation of c-*cerb*B-2 in human breast cancer. *Cell. Mol. Probes*, **2**, 283.

HEDLEY, D.W., FRIEDLANDER, M.L., TAYLOR, I.W., RUGG, C.A. & MUSGROVE, E.A. (1983). Method for analysis of cellular DNA content in paraffin-embedded pathological material using flow cytometry. *J. Histochem. Cytochem.*, **31**, 1333.

JANSSON, M., PHILIPSON, L. & VENNSTROM, B. (1983). Isolation and characterisation of multiple human genes homologus to the oncogenes of avian erythroblastosis virus. *EMBO J.*, **2**, 561.

KOUFAS, A., HAUSEN, M.F., LAMPKIN, B.C. & 4 others (1984). Loss of allel at loci on human chromosome 11 during genesis of Wilm's tumour. *Nature*, **309**, 174.

KRAUS, M.H., POPESCU, N.C., AMSBAUGH, S.C. & KING, C.R. (1987). Overexpression of the EGF receptor-related protooncogene *erb*B2 in human mammary tumour cell lines by different molecular mechanism. *EMBO J.*, **6**, 605.

LAND, H., PARADA, L.F. & WEINBERG, R. (1983). Tumourogenic conversion of primary embryo fibroblasts requires at least two cooperating oncogenes. *Nature*, **304**, 596.

MARIANI-CONSTANTINI, R., ESCOT, C., THEILETTE, C. & 4 others (1988). *In situ* c-*myc* expression and genomic status of the c-*myc* lucos in infiltrating ductal carcinoma of the breast. *Cancer Res.*, **48**, 199.

NISHIMURA, S. & SEKIYA, T. (1987). Human cancer and cellular oncogenes. *Biochem. J.*, **243**, 313.

PAGE, D.L., ANDERSON, T.J. & SAKAMOTO, G. (1987). In *Diagnostic Histopathology of the Breast*, Page, D.L. & Anderson, T.J. (eds), p. 193. Churchill Livingstone: Edinburgh.

QUIRKE, P., FOZARD, J.B.J., DIXON, M.F., DYSON, J.E.D., GILES, G.R. & BIRD, C.C. (1986). DNA aneuploidy in colorectal adenomas. *Br. J. Cancer*, **53**, 477.

SCHECHTER, A.L., HUNG, M.C., VAIDYAMATHAN, L. *et al.* (1985). The *neu* gene: an *erb*-B homologous gene distinct from and unlinked to the gene encoding the EGF receptor. *Science*, **229**, 976.

SCHWAB, M., ALITALO, K., KLEMPNAUER, K.-H. & 6 others (1983). Amplified DNA with limited homology to *myc* cellular oncogene is shared by human neuroblastoma cell lines and a neuroblastoma tumour. *Nature*, **305**, 245.

SEALY, L., PRIVALSKY, M.L., MOSCOVICI, G., MOSCOVICI, C. & BISHOP, J.M. (1983). Site specific mutagenesis of avian erythroblastosis virus: *erb*-B is required for oncogenicity. *Virology*, **130**, 155.

SEMBA, K., KAMATA, N., TOYASHIMA, K. & YAMAMOTO, T. (1985). A v-erbB related protooncogene c-*erb*B-2 is distinct from the c-*erb*B1/epidermal growth factor gene and is amplified in human salivary gland adenocarcinoma. *Proc. Natl Acad. Sci. USA*, **82**, 6497.

SLAMON, D.J., DEKERNION, J.B., VERMA, I.M. & CLINE, M.J. (1984). Expression of cellular oncogenes in human malignancies. *Science*, **224**, 256.

SLAMON, D.J., CLARK, G.M., WONG, S.G., LEVIN, N.J., ULLRICH, A. & MCGUIRE, W.L. (1987). Human breast cancer: correlation of relapse and survival with amplification of the HER 2/*neu* oncogenes. *Science*, **235**, 177.

TAVASSOLI, M. & SHALL, S. (1988). Transcription of the c-*myc* oncogene is altered in spontaneously immortalized rodent fibroblasts. *Oncogene*, **2**, 337.

THEILLET, C., LIDEREAU, R., ESCOT, C. & 5 others (1986). Loss of a c-H-*ras*-1 allele and aggressive human primary breast carcinoma. *Cancer Res.*, **46**, 4776.

VAN DE VIJVER, M., VAN DE BERSSEL, R., DEVILEE, P., CORNELISSE, C., PETERSE, J. & NUSSE, R. (1987). Amplification of the *neu* (c-*erb*B-2) oncogene in human mammary tumours is relatively frequent and is often accompanied by amplification of the linked c-*erb*A oncogene. *Mol. Cell Biol.*, **7**, 2019.

VAN DE VIJVER, M.J., PETERSE, J.L., MOOI, W.J. & 4 others (1988). *NUE*-protein overexpression in breast cancer. *N. Engl. J. Med.*, **319**, 1239.

VARLEY, J.M., SWALLOW, J.E., BRAMMER, W.J., WHITTAKER, J.L. & WALKER, R.A. (1987). Alteration to either c-*erb*B-2 (*neu*) or c-*myc* proto-oncogenes in breast carcinomas correlate with poor short-term prognosis. *Oncogenes*, **1**, 423.

VENTER, D.J., SANGEEV, K., TUZI, N. & GULLICK, W.J. (1987). Over expression of the c-*erb*B-2 oncoprotein in human breast carcinoma: immunohistological assessment correlates with gene amplification. *Lancet*, **ii**, 69.

VERMA, I.M., LAI, M-H.T., BOSSELMAN, R.A., MCKENNCH, M.A., FAN, H. & BERNS, A. (1980). Molecular cloning of unintegrated Moloney mouse sarcoma virus DNA in bactriphage h. *Proc. Natl Acad. Sci.*, **77**, 1773.

WEINBERGER, C., THOMPSON, C.C., ONG, E.S., LEBO, R., GRUOL, D.J. & EVANS, R.M. (1986). The c-*erb*A gene encodes a thyroid hormone receptor. *Nature*, **324**, 641.

YAMAMOTO, T., IKAWA, S., AKIYAMA, T. & 5 others (1986). Similarity of protein encoded by the human c-*erb*B-2 gene to epidermal growth factor receptor. *Nature*, **319**, 230.

YANG, J.K., MASTERS, J.N., ATTARDI, G. & 5 others (1984). Human dihydrofolate reductase gene organisation: extensive conservation of the G ± C rich 5'non-coding sequence and strong intron size divergence from homologous mammalian genes. *J. Mol. Biol.*, **176**, 169.

ZHOU, D., BATTIFORA, H., YOKOTA, J., YAMAMOTO, T. & CLINE, M. (1987). Association of multiple copies of the c-*erb*B-2 oncogene with spread of breast cancer. *Cancer Res.*, **47**, 6123.